# Design of a Functional Training Prototype for Neonatal Resuscitation

**DOI:** 10.3390/children1030441

**Published:** 2014-11-20

**Authors:** Sivaramakrishnan Rajaraman, Sona Ganesan, Kavitha Jayapal, Sadhani Kannan

**Affiliations:** Department of Biomedical Engineering, Sri Sivasubramaniya Nadar College of Engineering, Kalavakkam, 603 110 Tamil Nadu, India; E-Mails: sonatamilselvi@gmail.com (S.G.); kavithajayapalb@gmail.com (K.J.); sadhanikannan@gmail.com (S.K.)

**Keywords:** birth asphyxia, neonatal mortality, newborn care, neonatal resuscitation, force sensing resistor, compression count

## Abstract

Birth Asphyxia is considered to be one of the leading causes of neonatal mortality around the world. Asphyxiated neonates require skilled resuscitation to survive the neonatal period. The project aims to train health professionals in a basic newborn care using a prototype with an ultimate objective to have one person at every delivery trained in neonatal resuscitation. This prototype will be a user-friendly device with which one can get trained in performing neonatal resuscitation in resource-limited settings. The prototype consists of a Force Sensing Resistor (FSR) that measures the pressure applied and is interfaced with Arduino^®^ which controls the Liquid Crystal Display (LCD) and Light Emitting Diode (LED) indication for pressure and compression counts. With the increase in population and absence of proper medical care, the need for neonatal resuscitation program is not well addressed. The proposed work aims at offering a promising solution for training health care individuals on resuscitating newborn babies under low resource settings.

## 1. Introduction

**Figure 1:**
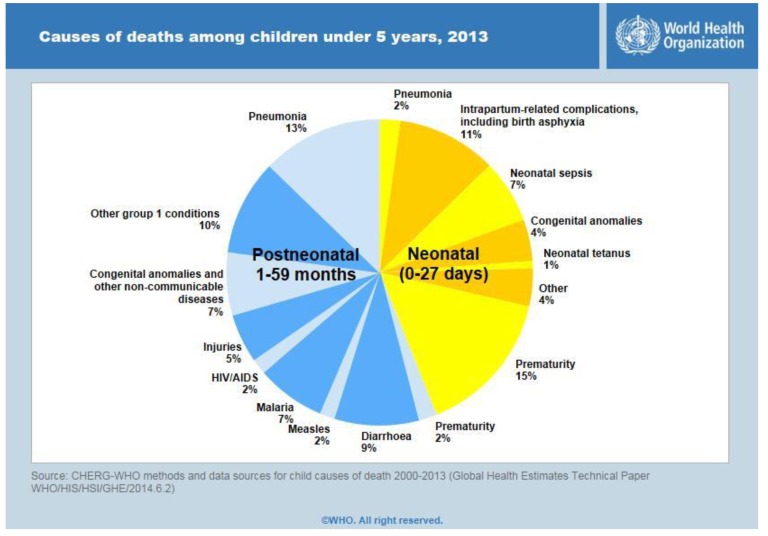
Statistics for causes of child mortality.

Neonatal Mortality Rate (NNMR) is defined as the number of infant deaths that occur in less than 29 days from birth for every thousand live births for a given year. The NNMR rate is rapidly increasing around the world and is the most prominent in rural regions. About 10% of neonatal mortality is caused due to birth asphyxia [1].The statistics for the causes of child mortality is shown in Figure 1. Asphyxiated neonates require skilled resuscitation to survive the neonatal period. Lack of trained medical staff, along with poor health infrastructures, is one of the major hurdles in ensuring quality neonatal care.

Neonatal mortality constitutes about 75% of infant deaths around the world. It is therefore essential that neonates be provided the utmost care at the first month of life so that neonatal deaths attributable to asphyxia, infection, and preterm births could be prevented [2]. The 2006 World Health Report stresses that the probability of infant, child and maternal survival is positively correlated with increasing density of competent health workers [3]. Despite a rapid economic growth that has occurred around the world over the last two decades, the neonatal mortality rate continues to remain high, around 900,000 in 2007, and India accounts for nearly 28% of the global deaths among newborn children [4]. Causes, including preterm delivery (29%), asphyxia (23%) and severe infections, such as sepsis and pneumonia (25%), account for three quarters of neonatal deaths around the world. Existing interventions can prevent two-thirds or more of these deaths if they reach those in need. Out of several million births each year, 4%–6% of neonates fail to establish spontaneous breathing at birth. Proper assistance can be provided to babies, if healthcare professionals present at the time of birth are skilled in the art of neonatal resuscitation [5]. There are more than a million neonatal deaths occurring in India every year and that accounts for almost a quarter of all the neonatal deaths across the world. As the quality of Cardiopulmonary Resuscitation (CPR) makes a huge difference between life and death, there is a huge demand for effective CPR training [6].

**Figure 2:**
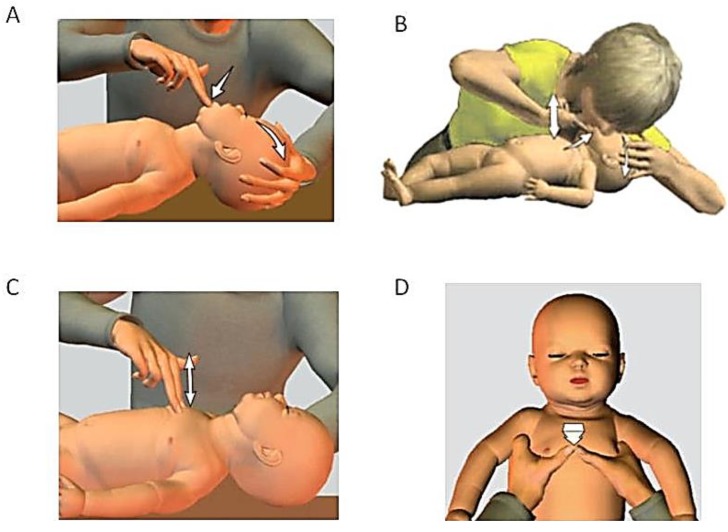
(**A**) Clearing the airway; (**B**) Providing assisted ventilation; (**C**) Two fingers method for compression; (**D**) Thumb encircling the chest method for compression.

CPR is a medical procedure that involves both compression and ventilation for the survival of a newborn. The first step involves clearing the airway. When the heart rate is less than 100 beats per minute, assisted ventilation must be provided using an endo-tracheal tube or a bag valve mask, called by its proprietary name as AMBU bag. When the heart rate is less than 60 beats per min, assisted ventilation along with chest compressions has to be performed. Chest compressions can be done in two ways: two fingers method and thumb encircling the chest method. Figure 2 depicts the steps involved in neonatal resuscitation. The goals of neonatal resuscitation are to prevent morbidity and mortality associated with hypoxic-ischemic tissue injury of the human organs and also to reestablish adequate spontaneous respiration and cardiac output [7].

When complications such as asphyxia or severe infection arise that lead to cessation of breathing and decrease of heart rate, it is difficult to maintain the right rhythm and correct ratio of insufflations to chest compressions and to exert the compressions at a constant pressure while performing CPR [8].CPR procedures are clearly described in the neonatal resuscitation guidelines of the American Heart Association [9]. When apnea persists and the heart rate is below 60 Beats per Minute (BPM), actual CPR should be instituted. CPR should be carried out with two persons, one performing insufflations and the other performing chest compressions. The ratio of insufflations and chest compressions should be 1:3. During the stress due to an acute situation, even for the medical staff, it is difficult to maintain the right rhythm and correct ratio of insufflations to compressions and to exert the compressions at a constant pressure [10]. On one hand, improper chest compressions could cause injury to the internal organs [11]. On the other hand, soft compressions could lead to lack of depth in compression, and, thus, it becomes mandatory to provide hands-on training to the attendants working on CPR. There are a few critical requirements for high-quality CPR that includes appropriate chest compressions at the exact depth and rate and to maintain a proper stature while doing compressions and avoiding disproportionate ventilation [12]. The quality of external chest compressions is the key component of basic life support. Different approaches are used to improve the performance that was evaluated using visual feedback [13]. The drawback of the method was an absence of indication for the compression count and audio feedback. The relationship between ventilation and chest compression was explained. There was an inverse relationship between intra-thoracic pressures and coronary perfusion pressures and subsequent survival from cardiac arrest [14]. Basic resuscitation would substantially reduce neonatal deaths. The practitioner needs to identify the delay between the intra and extra-uterine physiology and provide appropriate neonatal transition to support spontaneous breathing and cardiopulmonary functions [15]. When a baby is born in medical facilities, care must be taken to ensure that all birth attendants are competent in resuscitation. Innovative strategies to address the problems faced in home births are urgently required. Sufficient data are required to document the impact of neonatal resuscitation, particularly on long-term results in low-resource settings [16]. With the increase in population and absence of proper medical care, the need for Neonatal Resuscitation Program (NRP) is not well addressed.The Indian Academy of Pediatrics (IAP) provides NRP training to all the health care professionals who are involved in delivery or immediate interventions after delivery to provide appropriate medical care to the neonates [17].

The prototype reported in reference [8] provides a visual feedback with electroluminescent foil actuators for indication of the exerted chest compression pressure. It also provides audio feedback, which produces different sounds as audio guidance for providing ventilation and compressions but recording of the number of compressions and the pressure levels is performed by a separate evaluation mannequin embedded with sensors. However, the proposed technique helps to keep track of the pressure applied on the neonates and the number of compressions given per minute. The visual feedback provides the display for the pressure applied and the number of compressions given and helps in controlling the pressure if exceeded. The audio feedback gives an alarm if the pressure applied is very high.

The proposed work is aimed to design a functional training prototype for neonatal resuscitation to assist healthcare professionals in providing the most appropriate resuscitation to the neonates. The functional prototype is equipped with a visual and audio feedback that evaluates the performance of CPR. Special attention has been given towards its use in the most sensitive Neonatal Intensive Care Unit (NICU) environment, considering the power level, reliability of the sensors with respect to temperature fluctuations, and sensitivity to noise and heat variations. With the use of display and audio devices, the feedback on the performance of the healthcare trainee is received. Thus, this work aims to design a resuscitation tool that is specifically oriented to performing neonatal resuscitation in human resource-limited settings.

## 2. Materials and Methods

**Figure 3:**
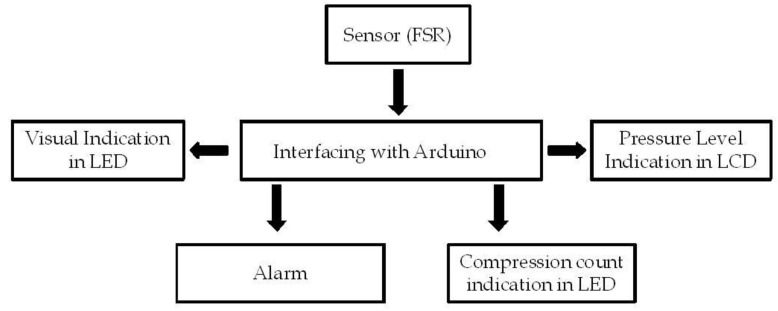
Block diagram of the prototype.

The schematic representation of the prototype is depicted in Figure 3. A Force sensing resistor (FSR) is used for measuring the applied pressure. FSR, known for its smaller size and low power consumption can be easily embedded into the system. The controller, to which the FSR is interfaced with, is Arduino^®^, an open source platform available for electronics prototyping for creating interactive environments [18].

**Figure 4:**
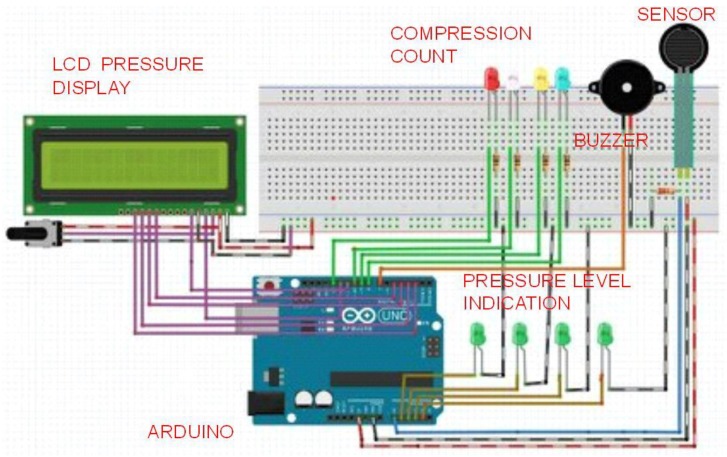
Overall prototype.

The FSR is connected through the voltage divider circuit. The pressure display unit consists of Liquid Crystal Display (LCD) that is connected to the digital ports of Arduino^®^. The pressure ranges are classified using the data obtained from the calibration procedure and displayed on the LCD. An alarm circuit is connected in parallel with the LCD and the buzzer goes off when the pressure exceeds the required level. The number of compressions per minute is provided by Light Emitting Diode (LED) indications. Different counts in the order of 15, 30, 60 and 100 are displayed in different colors. The prototype is tested with a mannequin and the results are validated. Figure 4 shows the overall prototype.

### 2.1. Arduino with ATmega8

Arduino^®^ is an open source prototyping platform based on simple hardware and software. The controller used is Arduino^®^ with an inbuilt low power CMOS 8-bit Atmega8 Microcontroller. The microcontroller has 8KB of in system programmable flash program memory that stores the program codes. It also includes 512Kb of EEPROM and 1Kb of SRAM. Atmega8 is a powerful microcontroller that provides a highly flexible and cost effective solution for many embedded control applications.

### 2.2. Pressure Display and Compression Count

**Figure 5:**
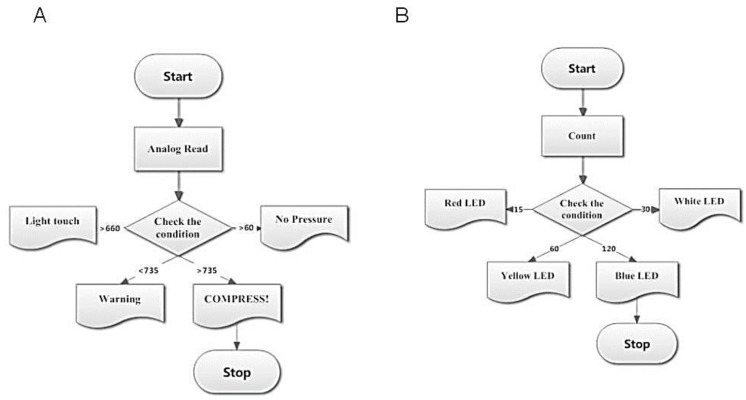
(**A**) Flowchart illustrating the flow of pressure display system; (**B**) Flowchart illustrating the compression count unit.

The program starts with the recording of analog readings obtained from the sensor output with which the applied pressure condition is checked. The pressure ranges are categorized depending on the analog values. Using these values, the algorithm is developed to display the pressure range on an LCD as shown in Figure 5A. During program compilation, the number of times the sensor is being compressed is counted. Depending upon the number of counts, indications are provided using different colors of LED. When the count reaches 15, 30, 60 and 100, the corresponding pins 7,8,13 and 10 go high and the red, white, yellow and green LEDs glow, respectively. The flow of the compression count unit is shown in Figure 5B.

### 2.3. Force Sensing Resistor (FSR)

**Figure 6:**
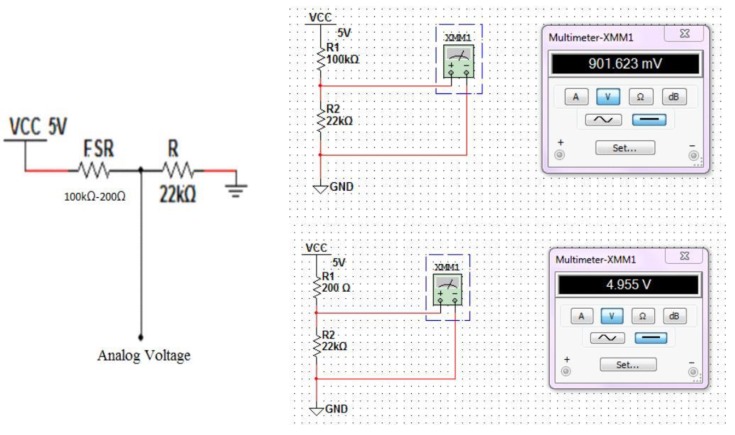
Voltage divider circuit.

FSR is a piezo-resistive polymer whose resistance changes when a force is applied. It exhibits a decrease in resistance with increase in force applied to the sensor. The force sensitivity range is between 0.1 and 100 N. The sensor is about 18.28 mm in diameter and 0.2 to 1.25 mm in thickness [19]. Figure 6 shows the voltage driver circuit. The sensor is connected to the Arduino^®^ using a voltage divider circuit. The voltage divider circuit provides the output voltage across two resistors connected in series. The FSR and the resistor form a voltage divider, which divides the supply voltage between the two resistances. The analog voltage “V_0_” is given by Equation (1):

V0 = Vcc (R / (R + value of FSR))
(1)

## 3. Results and Discussion

**Figure 7:**
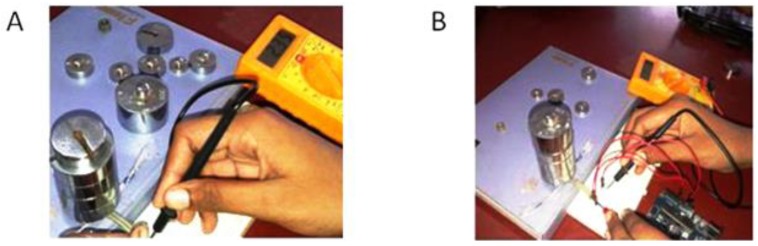
(**A**) Measurement of resistance; (**B**) Measurement of voltage.

The sensor and FSR are calibrated against known values of weights say 30, 35, up to 700 grams. The resistance and the corresponding voltage are measured using a digital multimeter as shown in Figure 7. Table 1 shows the resistance and conductance measured for different weights. The force was calculated as per Equation (2).

F=mg
(2)

Where “m” is the mass in kilograms and “g” is acceleration due to gravity (g = 9.8 ms^−2^). Table 2 shows the voltage measured for different weights. Table 3 displays the pressure values and the corresponding analog read values. The pressure is calculated from force using the diameter of the sensor. The values of resistance decrease with increase in weights as observed from Table 1. The values of voltage increase with increase in force but do not increase beyond 5 volts as observed from Table 2.

**Table 1:** Measurement of force resistance and conductance for varying values of weights.

Weight (in gms.)	Force (in N)	Resistance (in kΩ)
30	0.294	210.15
35	0.343	136
40	0.392	109.2
45	0.441	62
50	0.49	33.7
60	0.588	22.1
70	0.686	16.7
80	0.784	14.05
90	0.882	12.55
100	0.98	11.55
125	1.225	8.765
150	1.47	7
175	1.715	5.955
200	1.96	5.1
250	2.45	4.13
300	2.94	3.505
350	3.43	3.1
400	3.92	2.805
450	4.41	2.46
500	4.9	2.315
600	5.88	2.19
650	6.37	1.985
700	6.86	1.89

**Table 2:** Measurement of voltage for varying values of weights.

Weight (in gms.)	Force(in N)	Voltage(in V)
30	0.294	2.23333
35	0.343	2.40333
40	0.392	2.65333
45	0.441	2.79
50	0.49	2.9
60	0.588	3.08333
70	0.686	3.00667
80	0.784	3.22
90	0.882	3.45
100	0.98	3.57333
125	1.225	3.76
150	1.47	3.98667
175	1.715	4.15667
200	1.96	4.24
250	2.45	4.39667
300	2.94	4.47
350	3.43	4.5667
400	3.92	4.63
450	4.41	4.69
500	4.9	4.71
600	5.88	4.70667
650	6.37	4.71
700	6.86	4.71

**Table 3:** Pressure and the corresponding analog read values.

Analog Read	Force(in N)	Pressure(in N/m^2)
457	0.294	1121
492	0.343	1308
543	0.392	1494
571	0.441	1681
593	0.49	1868
631	0.588	2242
615	0.686	2615
659	0.784	2989
706	0.882	3362
731	0.98	3736
769	1.225	4670
816	1.47	5604
850	1.715	6538
868	1.96	7472
900	2.45	9340
915	2.94	11208
934	3.43	13076
947	3.92	14944
960	4.41	16812
964	4.9	18680
963	5.88	22416
964	6.37	24284

**Figure 8:**
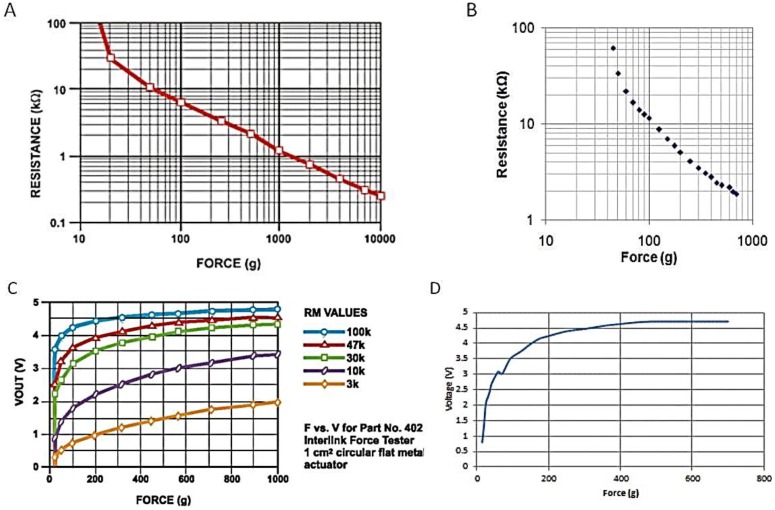
(**A**) Force *vs.* resistance obtained from datasheet; (**B**) Force *vs.* resistance obtained from calibration values; (**C**) Force *vs.* voltage obtained from datasheet; (**D**) Force *vs.* voltage obtained from calibration values.

**Figure 9:**
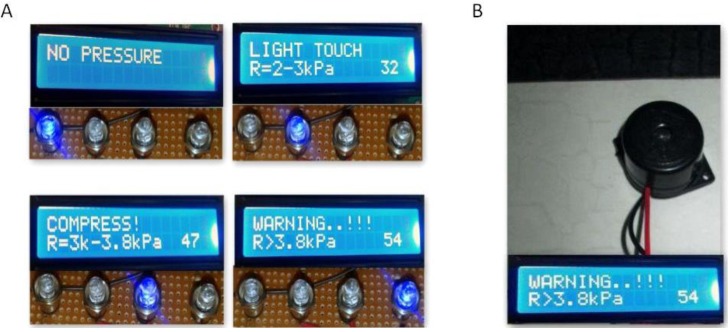

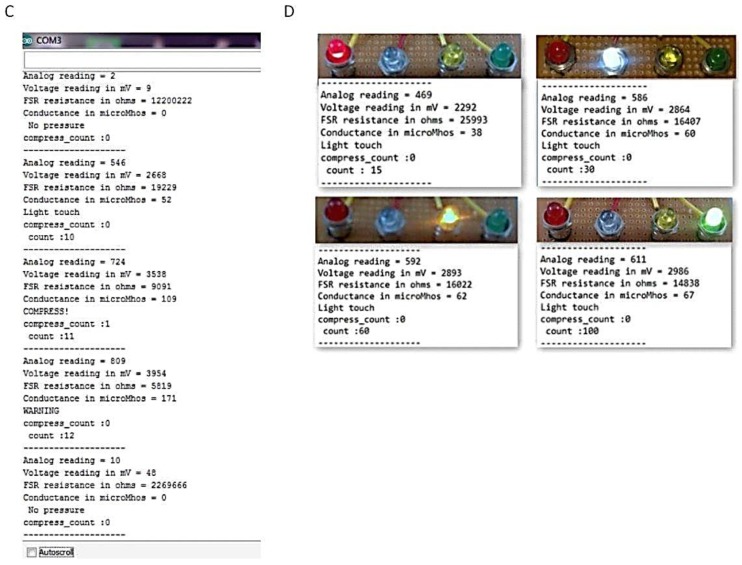
(**A**) Display for pressure ranges; (**B**) Alarm unit; (**C**) Output of serial monitor; (**D**) Compression count indication.

The normal pressure for neonatal resuscitation is about 30–40 cm of H_2_O [20] 2900–3900 N/m^2^. With reference to the normal pressure, the different pressures are categorized. The pressures less than this reference pressure are categorized as light pressures and pressures higher than the reference pressure are categorized as warning. With reference to this pressure, the pressure ranges are categorized and programmed using Arduino^®^. The prompts on the LCD are displayed according to the force sensed by the sensor. A visual feedback is given that provides the trainee, the necessary instructions during the procedure. Figure 8 shows a comparison of the plot of various values as obtained from the datasheet and from the calibration process. Figure 9A shows the LCD outputs of different pressure levels and their corresponding ranges. A buzzer connected to pin 9 of Arduino^®^ monitors the pressure level when the program starts running, and when the pressure level exceeds the required level, the buzzer goes off. Figure 9B depicts the alarm unit. The output from the serial monitor of Arduino^®^ is shown in Figure 9C. The combined rate of compressions and ventilation can be in the ratio 3:1 and this would provide 90 compressions and 30 breaths/minute [7]. Figure 9D shows the visual indication to keep track of the number of compressions given per minute.

## 4. Validation

**Figure 10:**
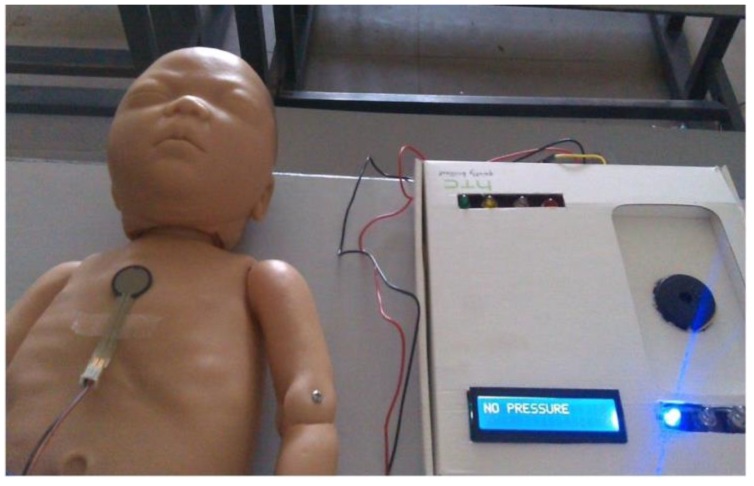
Testing prototype.

**Figure 11:**
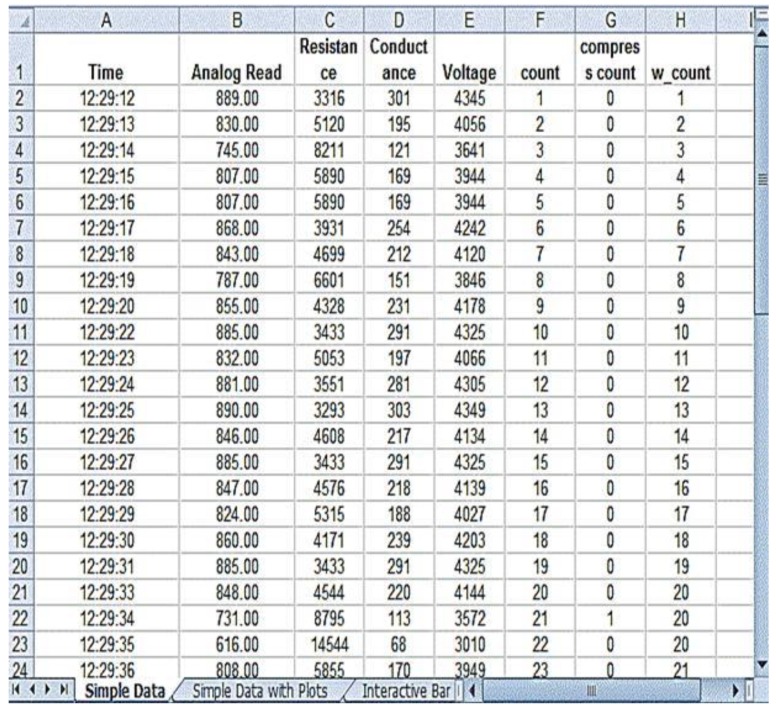
Without Feedback.

**Figure 12:**
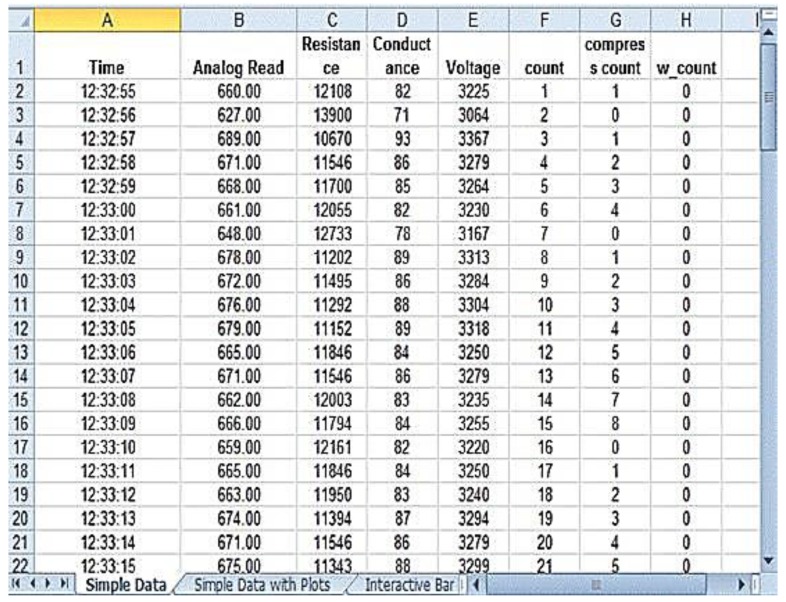
With Feedback.

**Figure 13:**
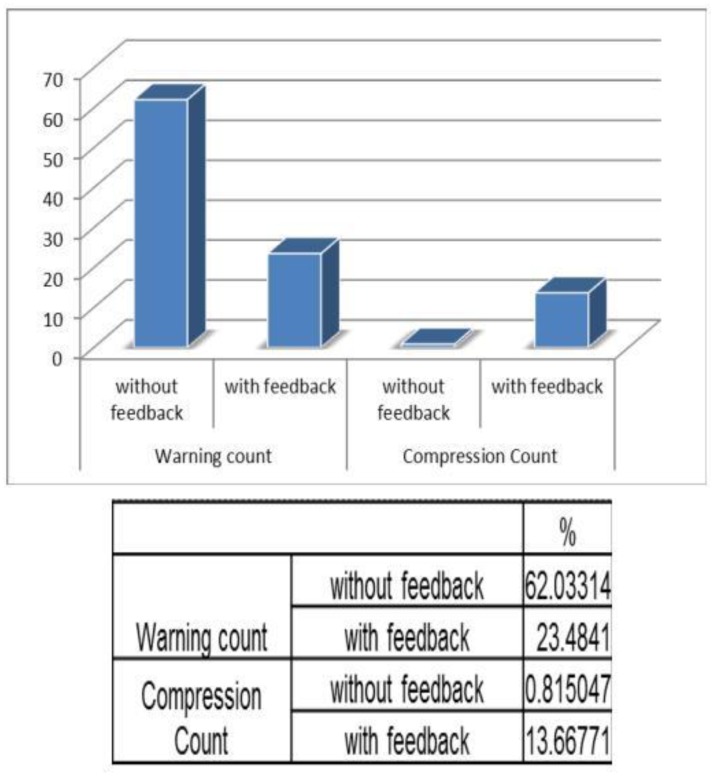
Validation chart.

The prototype used for validation of the results is shown in Figure 10. The prototype was validated in the Diagnostic and Therapeutic Equipment laboratory at the Department of Biomedical Engineering, Sri Sivasubramaniya Nadar College of Engineering, with 30 people doing CPR on the mannequin with and without visual and audio feedback. The ratio of ventilation to compressions was maintained at 1:3 for neonates; that infers that for a single ventilation, three compressions were done. The rate of chest compression used for validation was 90–100 per minute. During the Validation process, the trainee was asked to perform chest compression and the values, such as compression count and warning count were recorded in Excel. The Arduino was interfaced with PLX-DAQ supported spreadsheet which recorded the value during the process of chest compression. Figure 11 shows the PLX-DAQ spreadsheet and the values which were recorded during the validation process without feedback. Figure 12 shows the PLX-DAQ spreadsheet and the values which were recorded during the validation process with feedback. In Figure 11 and Figure 12, the column count refers to the total number of compressions given by the person; the column compress count refers to the number of required compressions *i.e.*, the pressure for performing CPR given by the person, and the column w count refers to the number of warning pressures provided. Figure 13 depicts the chart plotted based on the validation results. A graph is plotted using the obtained compression count and warning count and the comparison is studied.

It is observed that without feedback, the warning count percentage was about 66%. But with feedback, the percentage reduced to 30%. Similarly when the compression count is taken into account, the percentage increased from 1% to 3%, without feedback and with feedback, respectively. Hence, this prototype is a user-friendly device with which one can get trained in performing neonatal resuscitation in resource limited settings.

## 5. Conclusions

A prototype has been proposed to train health care professionals involved in the delivery of newborns or immediate interventions after delivery. The proposed technique helps to keep track of the pressure applied on the neonates and the number of compressions given per minute. Further, this prototype should be extremely helpful in Primary Health Centers (PHC) where there is inadequate manpower for training individuals. The work can be extended to measure more compression characteristics, including compression depth, frequency and acceleration. Further enhancements could be done by making the prototype wireless, self-powered and easily portable.
